# Supplementary material for: Are carbon emissions trading and green financial instruments synergistic? -Comprehensive quantitative research based on content analysis
Source: PLoS One. 2024 Mar 7;19(3):e0298601. doi: 10.1371/journal.pone.0298601 (PMC10919601; doi:10.1371/journal.pone.0298601)
Supplement: S1 Data — (ZIP) [file pone.0298601.s001.zip › Data/地级市Z_F工作报告.zip!/╜¡╬≈/╛┼╜¡╩╨/└┤╫╘╣1⁄2╓┌║┼í╛┬φ┐╦╩2╛▌═°í┐_2016.docx]

各位代表：

　　现在，我代表市人民政府，向大会报告工作，请予审议，并请各位市政协委员和列席会议的同志提出意见。

　　一、“十二五”及2015年工作回顾

　　“十二五”时期，是九江历史上发展最快、变化最大、群众得到实惠最多的时期之一。五年来，在省委、省政府和市委的正确领导下，全市上下围绕“做大九江、打造双核”的目标定位，主动适应新常态，始终保持实干定力，努力争得九江应有地位，经济社会保持了良好的发展态势，为“十三五”全面建成小康社会奠定了坚实基础。

　　——五年来，经济总量持续扩张，发展质量得到新提高。生产总值由1032亿元增加到“十二五”末的1902.7亿元，年均增长11.1%；财政总收入由116.7亿元增加到385.6亿元，年均增长27%；公共财政预算收入由71.1亿元增加到247.2亿元，年均增长28.3%；固定资产投资由722.2亿元增加到2119.9亿元，年均增长24%；社会消费品零售总额由279.1亿元增加到578.6亿元，年均增长15.7%；居民消费价格总水平保持平稳。三次产业比例由9.5∶56.2∶34.3调整为7.4∶53.3∶39.3。县县财政过10亿元，7个县财政过20亿元。

　　——五年来，发展平台加速拓展，区域格局实现新优化。永武高速、都九高速（星子至九江段）建成通车，新增高速公路通车里程193公里，实现县县通高速；九江二桥和105、316国道（九江段）、共安大道等建成通车，改造干线公路705公里，新建改造农村公路7328公里。完成电网投资66.4亿元，500千伏石钟山至洪源线路，220千伏码头、沙城扩建等一批输变电设施投入使用。沿江“1+5”发展规划对外发布，累计完成基础设施投入660亿元，一批区间通道、疏港通道建成通车；新建码头泊位28个，长江港口货物吞吐量实现三年翻番，提前五年建成亿吨大港。全市工业园区完成基础设施投入735亿元，总面积突破100平方公里。12个省级以上工业园区主营业务收入均超百亿元，九江经济技术开发区突破1000亿元，“沿江挑大梁、沿路做支撑、县区为依托”的发展格局基本成型。

　　——五年来，强工氛围更加浓厚，工业发展实现新突破。全市规模以上工业增加值由360.6亿元增加到1035.6亿元，年均增长13.7%；主营业务收入由1549亿元增加到5109.2亿元，年均增长27%；利税总额由163.9亿元增加到570.1亿元，年均增长28.3%。规模以上工业企业净增407户，达1259户，总量全省第一。十大主导产业加速集聚，新兴产业加快发展，9个特色产业集群入选省重点调度名录。九江经济技术开发区获批国家船舶配套设备高新技术产业化基地，庐山区获批国家玻璃纤维及复合材料高新技术产业化基地，共青纺织服装、永修有机硅创建国家新型工业化示范基地。

　　——五年来，兴城步伐不断加快，城乡面貌发生新变化。中心城区从“两湖”时代迈进“八里湖”时代，建成区面积由80平方公里扩大到103平方公里。成功创建国家园林城市、全国无障碍建设先进城市。八里湖新区实施城建项目170个，一批基础设施、文化旅游、社会事业项目交付使用，一座功能日趋完善、人气不断提升的现代化生态新城基本成型。老城区改造步伐加快，完成棚户区、城中村改造600多万平方米，整治改造边街小巷345条，改造提升主次道路43条，人均公园绿地面积17.6平方米。绕城高速、九码快速通道等建成通车，“四县一山”加快融入中心城区。县城建设投入不断加大，一批规划科学、配套齐全、环境优美的县城新区建成使用。集镇功能不断完善，镇村联动、新农村建设、“两违”整治和“两沿”提升等工作扎实推进，农村面貌明显改善。全市城镇化率提高8个百分点。

　　——五年来，农业经济平稳发展，基础地位得到新增强。粮食实现“十二连丰”，油菜、棉花播种面积和总产量均居全省第一。流转耕地144.8万亩、林地184.7万亩，创建省级农业产业集群10个、现代农业示范区9个，赛湖农场获评国家农业综合开发现代农业示范园，共青城获评国家农业产业化示范基地。新增规模以上农业企业204家。创建“三品一标”324个、省级以上农产品品牌128个，“双井绿”“彭泽鲫”被核准为国家地理标志证明商标。国家农业综合开发县、小农水重点县实现全覆盖，建设高标准农田40万亩，完成913座中小型水库除险加固、43条中小河流治理。改造灌区741处，新增、恢复和改善灌溉面积170万亩。

　　——五年来，对外开放升温加热，改革创新取得新成效。全市累计开工市外5000万元以上项目1859个，实际进资3207.9亿元。实际利用外资由6.7亿美元增加到16.3亿美元，年均增长19.6%；进出口总额由18.2亿美元增加到60亿美元，年均增长26.9%；境外直接投资由1237.4万美元增加到9988.9万美元，年均增长68.6%。集体林权制度改革和基层农技推广体系改革的经验在全国推广。在全省率先开展农村集体土地承包经营权抵押贷款试点，国企、农垦企业和国有林场改革全面完成。政府机构、户籍制度、投融资、文化、医药卫生体制等改革深入推进。创新了土地清理、河道采砂等管理体制。新增省级以上高新技术企业83家，新建院士工作站19家，新增中国驰名商标6个，专利申请量8325件，授权量5233件。昌九两地达成六大类25个合作事项，公交、通信、基本医疗保险、户口迁移等合作顺利完成。共青城列入省直管县试点，体制机制创新成效明显。新增4A级景区14个，旅游经济主要指标总量连续五年居全省首位。

　　——五年来，环境保护力度加大，生态建设厚植新优势。生态文明先行示范区建设扎实推进，基本完成“十二五”节能减排任务。新增国家级生态乡镇27个，建成国家级自然保护区3个、湿地公园3个、森林公园8个。PM2.5实现24小时在线监测，空气环境优良天数年均保持在310天以上。完成造林绿化161.6万亩，森林覆盖率55.2%。8个县成功创建省级森林城市。建成水质自动监测站8个，境内长江、修河监测断面水质达III类水标准，集中式饮用水源地水质达标率100％。新增农村安全饮水人口155万人，治理水土流失180万亩。修水获评国家水土保持先进县。永修云山经济开发区通过国家循环经济试点验收。

　　——五年来，民生投入不断增长，人民生活有了新改善。全市累计用于民生领域的财政支出突破1000亿元。城镇居民人均可支配收入由15764元提高到27635元，农村居民人均可支配收入由5588元提高到11143元。建成各类保障房33.2万套，新改建农村危旧房5.6万户。新增城镇就业32万人，转移农村劳动力30.9万人。中心城区新建改造农贸市场16个、停车场8个、公交综合场站7个，新增公交车313辆。创建“放心粮油”示范点145个，建成农家店317家、配送中心12家、乡镇商贸中心23个，新建城郊蔬菜基地2.5万亩，新增粮食库容26万吨。完成教育投入296.8亿元，新改扩建校舍总面积189万平方米，补充教师7737名；九江职业大学、九江职业技术学院、庐山区第一中学、庐山区中心幼儿园、科技中专、九江一中、九江小学、双峰小学等新校区建成使用；新增公办幼儿园512所，学前教育三年入园率达84.2%；义务教育均衡发展，高中教育、职业教育、特殊教育和高等教育协调发展。在全国率先实施新农合跨省跨市直补、先看病后付费等新模式，城乡基本医疗保险和基层医疗服务网络实现全覆盖。全面实施城乡困难群众医疗救助，贫困家庭重大疾病免费救治惠及5.9万人，血吸虫病疫区均达到传播控制以上标准。文化惠民工程扎实推进，公共文化设施实现县乡村全覆盖，“四馆一站”免费开放。体育产业和竞技体育加快发展，全民健身活动蓬勃开展。全国食品药品安全示范区建设扎实推进，食品药品科学监管水平不断提高。大力实施精准扶贫，减少贫困人口21.3万人，安置搬迁移民8.4万人，修水、武宁扶贫移民整体搬迁模式在全国推广。第九届村（居）委会换届选举全面完成，基层政权建设和民主管理进一步加强。安全生产形势总体稳定，事故总起数和总死亡人数实现双下降。社会治安形势更加平稳，信访秩序明显好转，公众安全感明显提高，社会保持和谐稳定。

　　2015年主要抓了以下八个方面工作：

　　（一）深入推进重大战略。沿江开放开发扎实推进，九江港总体规划修编完成，省煤炭储备中心、码头工业城等港口码头项目基本完工，九景衢铁路、武九客专、城东港区铁路专用线等项目进展顺利。省政府支持昌九一体化发展20项政策措施加快落实，实施“飞地”合作项目16个。昌九高速“四改八”（通远段）建成通车，都九高速（都昌至星子段）开工建设，昌九大道全线贯通。共青科教城、共星大道等项目快速推进，青年电商产业园、APP孵化产业园建成运营，私募基金创新园区加快发展。创建国家森林城市步伐加快，庐山大道、长虹大道、九龙山森林公园等绿化改造提升基本完成，八里湖绿道系统等项目加快建设。城市水生态文明建设规划编制完成，白水湖截污工程等项目加快推进。柘林湖湖泊生态环保PPP示范项目开工建设，15个百强中心镇生活污水处理设施建成运营。鄱阳湖湿地列入生态补偿政策试点，武宁入选全国生态保护与建设示范区，德安获评国家园林县城，星子列为全省非金属矿山综合整治试点县。

　　（二）全力以赴决战工业。规模以上工业增加值同比增长9.9%，主营业务收入增长7.5%，利税总额增长13.2%。全年四批次集中开工项目420个，九江石化油品质量升级、瑞智机电等竣工投产，神华九江电厂、兄弟医药、九宏新材料等加快推进。全市工业园区建成公共服务信息平台8个，开展“两化融合”试点企业60户，九江石化智能工厂列入全国试点，永修云硅创建全国首家有机硅产品电商平台。瑞昌、星子、湖口等工业园完成扩区调区规划。出台决战工业一万亿“1+2”文件、促进经济平稳健康发展“55条”、推进大众创业万众创新“28条”等政策措施。设立了总规模50亿元的市政府产业投资引导基金，通过财园信贷通、助保贷等模式有效缓解企业融资难问题。盛祥电子、禾益化工、美凯宝挂牌“新三板”。

　　（三）着力提升门户形象。中心城区实施城建项目187个，完成投资158亿元。城市总体规划获批，中心城区、县城控制性详细规划和乡（镇）总体规划、行政村规划实现全覆盖。三马路、考棚路和战备路等片区改造加快推进，通岭大道、兴城南大道、市中心幼儿园八里湖校区、九派诗廊等新区功能项目加快推进，国际金融广场、九江茶市、万达广场、京九商贸城、禧徕乐国际商贸中心等城市综合体项目加快推进。扎实开展城市环境综合整治“百日大会战”和“攻坚年”活动，垃圾死角、建筑工地、占道经营、交通秩序等专项整治成效明显，城市管理长效机制逐步健全。

　　（四）繁荣发展第三产业。第三产业实现增加值747.3亿元，增长11.5%。旅游产业创新发展规划编制完成，庐山设市工作全面启动。庐山西海国际生态旅游度假区、归宗旅游度假综合体等项目加快推进。庐山核心景区实现“一票制”。“庐山藏天下，九江通四海”城市形象营销向全国推广。庐山获评中国最具价值文化（遗产）旅游目的地景区，庐山西海获评全国十佳生态旅游示范景区，星子获评中国最佳文化生态旅游名县。全市旅游接待总人数突破1亿人次，增长40%；旅游总收入922亿元，增长48%。新增物流企业151家，完成集装箱吞吐量25.5万标箱，公路货运量1.2亿吨，货运周转量266亿吨公里。全市电子商务实现销售额255.3亿元，阿里巴巴九江产业带入驻商户555家，星子横塘镇获评省电子商务示范基地。光大银行、北京银行、恒邦财险、中信证券等落户九江，全市金融机构本外币各项贷款余额1460.7亿元，增长18.7%。总部经济、楼宇经济、家政服务和社会养老服务等业态蓬勃发展。

　　（五）大力夯实三农基础。新建千亩以上农产品基地73个，新增农民合作社797家、家庭农场648家。茶叶品牌建设成效显著，在国内外荣获多项大奖。831个新农村建设点、87个中心村建设有序推进，改水、改厕3.9万户。农村生活垃圾治理全面启动，解决25万农村人口饮水安全问题。发放粮食补贴3亿元，发放“财政惠农信贷通”贷款13.9亿元。完成“新型职业农民培育”5630人、“雨露计划”培训5285人，“一村一名大学生工程”招生900人，转移农村劳动力6万人。整村推进395个贫困村村庄整治，建成扶贫生态示范村100个。

　　（六）全面深化改革创新。严格落实“三单一网”改革要求，市县政府权力清单、责任清单挂网公布，九江政务服务网上线运行。市本级行政审批事项精简率达64.6%，全面取消非行政许可审批事项178项，清理取消行政审批前置中介服务53项。行政服务标准化试点工作通过验收。农村集体土地确权登记颁证工作基本完成，行政机关公车改革稳步推进。工商、质监、食品、药品、卫生、计生等机构改革全面完成。商事制度改革加快推进，“三证合一”“一照一码”全面实施，新增企业1.2万户，新增个体工商户3万户。九江港口岸扩大开放城西港区通过国家验收，城西港纳入全国启运港退税政策试点，九江海关纳入全国通关一体化；获批进口粮食指定口岸，进境木材监管区建成运营。瑞昌获批国家知识产权试点城市，恒盛科技园获批国家知识产权试点园区，津晶城科技园获评国家级科技企业孵化器。

　　（七）持续保障改善民生。财政性民生支出351.8亿元，占总支出79.4%，省下达民生工程任务全面完成。创新开展棚户区改造货币化安置和保障房“以购代建”工作，率先在全省实施保障房“租售并举”、物业管理新模式。新增城镇就业5.4万人，为城乡困难群众购买公益性就业岗位4400个。新增小额担保贷款14.9亿元，扶持创业1.4万人。城乡低保、农村五保集中供养和失业保险标准每人每月分别增加20元、30元和255元。城乡居民基础养老金和企业退休人员养老金每人每月分别增加25元、285.2元，环卫工人工资标准和社区干部工资待遇进一步提高。中心城区新增新能源公交车90辆、公交线路5条。农村义务教育学校标准化建设扎实推进，职业教育资源加快整合，高考成绩创十年最佳。“八个一”文化亲民工程加快实施，新增国家一级文化馆3家、图书馆6家。取消县级公立医院药品加成，分级诊疗体系和全国精神卫生综合管理示范区启动建设，新农合参合率达98.6%。“单独两孩”政策稳妥实施，人口自然增长率稳定在7.6‰以下。成功举办第七届老年人健身体育运动会。安全生产形势进一步好转，一批信访突出问题得到解决。深入开展治安、禁毒等专项行动，严厉打击各种违法犯罪行为，公众安全感和群众满意度不断提高。统计、审计、气象、水文、盐业、通信、无线电管理、档案、司法、国安、保密、机构编制、地方志、对台、外事侨务、民族宗教、驻外联络等工作得到新加强；国防动员、民兵预备役、双拥创建、人民防空、防震减灾、应急管理、武警、消防等工作取得新成绩；妇女儿童、老龄、残疾人、志愿者、红十字和慈善等事业实现新发展。

　　（八）自身建设不断加强。认真落实中央“八项规定”和国务院“约法三章”，扎实开展“三严三实”专题教育，全市政府系统干部作风明显好转。自觉接受人大依法监督、政协民主监督和社会舆论监督。制定出台《法治九江建设纲要》，深入推进政务公开和政府信息公开，办理市人大代表建议170件、市政协提案363件，办结率100%；市政府民声直通车受理和办理群众意见、建议、诉求8529件，办结率95.3%。积极巩固“红包”问题治理成果，深入开展领导干部违规插手干预工程项目问题专项整治，坚决查办了一批违纪违法腐败案件。“三公”经费支出同比下降9.4%。

　　需向代表说明的是，随着我国经济发展进入新常态，增速由高速转向中高速，“十二五”中后期全省经济发展增速普遍放缓，经济下行压力加大。对照“十二五”规划目标，我市也有少数指标低于预期，GDP、社会消费品零售总额年均增幅分别低1.9和2.3个百分点。总体看，我市“十二五”规划目标任务基本完成，九江发展站在了新的历史起点上。

　　各位代表，回顾“十二五”，我们坚持科学发展、跨越发展，历程极不平凡，经验弥足珍贵。我们深切地感受到：成绩的取得，是省委、省政府和市委正确领导的结果，是市人大依法监督、市政协民主监督和社会各界大力支持的结果，是全市人民团结奋斗、顽强拼搏的结果。在此，我代表市人民政府，向全市广大干部群众，向各位人大代表、政协委员、各民主党派、工商联、无党派和社会各界人士，向驻浔单位、人民解放军、武警官兵、公安干警和离退休老同志，向所有关心、支持、参与九江建设发展的朋友们，表示衷心地感谢并致以崇高地敬意！

　　我们深刻地体会到：做好政府工作，必须保持政治定力不偏向，在市委的正确领导下，不分心、不观望、不折腾，一心一意干事业，凝心聚力促发展。必须咬定发展目标不动摇，坚持目标引领，强化责任倒逼，一任接着一任干，一张蓝图画到底。必须突出工作重点不放松，坚持发展第一要务，保持经济增长中高速，加快做大经济总量，提升发展质量和效益。必须强化实干作风不懈怠，求真务实，真抓实干，敢于担当，勇于创新，善于破难，不断提高工作成效。必须牢记为民宗旨不淡忘，把人民满意作为最高追求，把实事办实，把好事办好，着力提升人民群众的幸福感和获得感。

　　我们清醒地认识到：经济总量不大、发展不足，加快发展的任务仍然艰巨；经济质量不高，产业结构不优，第三产业比重偏低，提质增效、转型升级的任务仍然艰巨；县域经济整体实力不强，在全省争先进位的任务仍然艰巨；城镇化水平不高，加快推进新型城镇化的任务仍然艰巨；群众收入偏低、部分居民生活还比较困难，同步全面小康的任务仍然艰巨；影响和谐稳定的因素依然存在，加强和创新社会管理的任务仍然艰巨；发展环境有待进一步优化，依法行政水平有待进一步提高，履职担当的干部作风有待进一步加强。对此，我们将采取更加有力的措施，积极应对，努力解决。

　　二、“十三五”时期经济社会发展总体安排

　　“十三五”时期，和平、发展、合作、共赢仍是时代主题，世界经济在深度调整中曲折复苏，我国经济长期向好的基本面没有改变，我省仍处于大有作为的重要战略机遇期，我市经济社会发展突出表现为“五期交织”的阶段性特征，即：率先全面小康的决胜期、全力做大九江的决战期、经济发展的转型期、发展动力的转换期、区域竞争的洗牌期。未来五年，九江发展的机遇更加难得、动力更加充足、基础更加稳固、氛围更加浓厚，全市上下务必科学认识新常态、主动适应新常态、积极引领新常态，保持战略定力、实干定力，构筑发展新优势，开创发展新局面！

　　全市经济社会发展的指导思想是：高举中国特色社会主义伟大旗帜，以邓小平理论、“三个代表”重要思想和科学发展观为指导，深入贯彻习近平总书记系列重要讲话精神，落实中央“五大发展”理念和省委“十六字方针”，以“推进‘T’型崛起，打造山水名城，率先全面小康，建设五大九江”为发展战略，聚焦“一心两翼三板块”，实施“新工业十年行动”，协同推进“五化”，把九江打造成为全省绿色崛起的双核之一、长江经济带重要中心城市、世界知名的山水文化名城和旅游度假胜地。

　　“十三五”全市经济社会发展主要目标是：生产总值年均增长10%左右；财政总收入年均增长11%；固定资产投资年均增长17%；社会消费品零售总额年均增长11%；规模以上工业增加值年均增长10%以上；城镇化率达到56%；城镇居民和农村居民人均可支配收入年均分别增长10.5%和12%；完成省下达的节能减排任务。

　　为如期实现“十三五”奋斗目标，我们将通盘统筹、科学谋划，加快构建“一心两翼三板块”的经济格局。做美“一心”，优化城市空间布局。以中心城区为核心，以卫星城市为支撑，以庐山为后花园，促进港城联动、产城融合、景城一体，着力构建“揽山入城、拥江抱湖”的九江都市区，力争“十三五”末中心城区人口达100万，中长期建成区面积达300平方公里，打造宜居宜游九江。做强“两翼”，优化区域空间布局。以东部湖区和西部山区为“两翼”，在保护中发展、在发展中保护，在扶贫中发展、在发展中扶贫，打造现代农业、绿色工业、生态旅游为代表的生态经济带。做实“三板块”，优化产业空间布局。以湖口县、彭泽县为主体的沿长江东部板块，重点建设长江经济带产业转移升级集中区，积极承接长三角、珠三角产业转移，加快推动化工、钢铁、纺织等传统产业转型升级；以九江经济技术开发区、瑞昌市、庐山区、九江县为主体的沿长江中西部板块，重点发展战略性新兴产业、高新技术产业和现代制造、港口贸易等中高端产业；以共青城市、德安县、永修县为主体的沿昌九南部板块，重点建设全省的“双创”基地，加快发展高新技术产业。

　　未来五年，将重点实施八大行动：

　　一是实施“新工业十年行动”，全力推动工业经济转型升级。大力推进新型工业化，对接“中国制造2025”，促进工业经济量质并举、结构升级、效益提升，加速实现规模以上工业主营业务收入万亿目标。实施传统产业提升计划。以石油化工、钢铁有色、建材、纺织等产业为重点，支持企业加快技术改造、重组整合、股改上市，推动传统产业改造升级。实施新兴产业倍增计划。以新能源、新材料、电子电器、生物医药、绿色食品和装备制造等产业为重点，通过引进重大项目、加快科技创新、完善扶持政策，推动战略性新兴产业向高端化、集约化、特色化发展，努力实现产业规模倍增、龙头企业倍增、示范基地倍增。实施主导产业集聚计划。突出龙头带动，延伸产业链条，推动产业集聚集群集约发展，着力提升产业规模和竞争力。坚持数字化、产业化、智能化方向，加快推进工业化和信息化深度融合，创建一批“两化融合”示范园区和企业。全力推动九江经济技术开发区做大做强，大力支持瑞昌申报国家级经开区、共青城申报国家级高新区。

　　二是实施“开放升级行动”，着力构筑内陆双向开放的前沿阵地。积极策应国家“一带一路”、长江中游城市群、长江经济带建设等重大战略，充分放大沿江临港优势，不断提升对内对外开放水平，打造开明开放九江。提升开放平台。推进九江综合保税区审批建设工作，积极申报整车、肉类、冰鲜水产品、水果等特定进口商品指定口岸；加快电子口岸建设，实现“三互”大通关，推动九江港开放升级。积极参与沿江经济协作区建设，大力承接发达地区先进制造业和先进技术转移。深入推进昌九一体化，以共青先导区为核心，推动昌九新区建设，加快打造江西融入长江经济带的“桥头堡”。提高招商引资水平。坚持把招商引资作为“头号”经济工程，实施精准化招商、精细化服务和精确化考核。抓住国家政策调整、产业重新布局的发展机遇，瞄准国内外大企业，招引一批产业带动性强、技术含量高的重大项目。坚持产业招商、专业招商、领导招商、以商招商、资本招商和环境招商，优化产业承接和项目配套服务，实现招商由单向承接向双向互动转变。完善客商服务机制，实施准入前国民待遇加负面清单管理制度，优化营商环境。积极推动“走出去”。健全走出去政策支撑体系，鼓励有条件的企业到境外投资发展，支持优势产业参与国际产业分工，深度融入全球产业链、价值链和物流链。

　　三是实施“创新创业行动”，着力促进发展动力转换。坚持以供给侧结构性改革为主线，大力推动大众创业万众创新，集聚发展新动力，打造创新创业九江。促进全方位改革。发挥市场在资源配置中的决定性作用，进一步完善市场体系，优化市场环境，激活市场主体，实现资源配置效益最大化和效率最优化。认真落实、完成中央和省委、省政府部署的改革任务，深入推进行政管理、审批制度、农业农村、财税金融等各项改革。主动先行先试，扎实开展一批具有九江特色的改革试点。支持多层次创新。加大知识产权保护力度，充分发挥企业主体作用，加强产学研对接，培育一批科技领军人才，打造一批科技创新平台，建设一批科技成果转化基地。加快智慧九江建设，统筹实施“互联网+”行动，促进信息化与经济社会各领域的深度融合发展。加快品牌创新，实现“九江制造”向“九江创造”转变。鼓励多渠道创业。完善创业扶持政策，构建低成本、便利化、开放式的众创平台。鼓励支持本土人才大胆创业、成功人士回归创业、务工人员返乡创业，打造一批创业示范基地。

　　四是实施“名城创建行动”，着力打响大山大水大九江的独特品牌。突出山水特色，完善城市功能，挖掘文化内涵，留住城市记忆，提升城市品位，把九江建设成为生活宜居、生态优美、生产繁荣的山水文化名城。彰显城市宜居特质。加快构建九江都市区，启动主城区快速通道和九江都市区轻轨建设，实现基础设施互联互通。做优做美中心城区，高标准打造八里湖新区中央商务区，规划建设高铁新区、产城融合试验区，推进赛城湖片区与八里湖新区对接融合。加快棚户区改造、危房改造、老旧小区改造步伐。积极创建国家历史文化名城、国家卫生城市、国家森林城市和全国文明城市。彰显城市宜游特质。推进国家级旅游度假区建设，优化精品线路，丰富旅游业态，提升旅游产业，加快旅游转型升级。以庐山管理体制改革为契机，整合优势资源，强化区域协作，着力构建“环庐山、大庐山、泛庐山”和庐山西海旅游经济圈，沿长江和鄱阳湖旅游经济带，打造“两圈两带”大旅游格局，推进旅游发展全域化、旅游产业全链化。彰显城市宜商特质。坚持以产促城、以城兴产、产城融合，加快发展现代金融，积极引导各类金融组织和服务机构入驻。加强政府性融资平台建设，加快企业上市步伐。发展金融要素市场，建立多层次资本市场，逐步健全现代金融体系，积极打造赣鄂湘皖四省边际区域金融中心。加快发展商贸物流，规划建设一批现代物流园区，培育引进一批重点物流企业，健全城乡商贸物流服务体系，加速形成以港口为中心的物流网络，打造连接长三角、辐射湘鄂皖、服务全省的物流枢纽。大力发展电子商务，积极发展健康养老、文化创意、工业设计、家庭服务等新型业态。

　　五是实施“生态优先行动”，着力打造生态文明“九江样板”。坚持绿色富市、绿色惠民，加快生态文明先行示范区建设，走生态优先、绿色发展之路，建设天蓝、地绿、水净的美好家园。保护生态环境。坚持预防为主、源头控制、综合治理，实行最严格的环境保护制度。深入实施“净空、净水、净土”行动，健全实时在线环境监控系统，完善环境监督保护机制，形成政府、市场、公众多元共治的环境治理体系。提升生态优势。合理划定并严守生态红线，构建沿长江、环鄱湖、覆盖庐山的生态安全格局。实施人工造林和封山育林，加强公益林保护，提升森林资源质量。加强水土流失综合防治，强化饮用水源保护区、水源涵养区、江河源头区的保护与生态修复。发展生态经济。探索“绿色+”产业发展模式，构建环境友好的绿色工业、生态有机的绿色农业和集约高效的绿色服务业体系。优化配置土地资源，集约利用岸线资源，科学开发矿产资源，提高资源利用效率。

　　六是实施“基础提升行动”，着力增强发展支撑功能。坚持统筹规划、适度超前，加快构建综合立体、功能配套、安全高效的现代化基础设施体系。强化交通支撑。积极对接沿长江综合运输通道建设，以高等级航道、疏港通道、港区建设为重点，以公路、铁路、航空、管道运输为支撑，构建便捷高效、联江通海的现代化立体交通格局，打造长江中游城市群重要交通枢纽城市。强化能源支撑。实施清洁煤电工程，发展新能源和可再生能源，尽早启动彭泽核电站建设。以500千伏、220千伏网架为支撑，支持1000千伏特高压建设，构建覆盖全市的环状电力骨干网架。推进新一轮农村电网、配电网改造升级，不断完善能源保障体系。强化水利支撑。争取长江干堤升级整治和江新、棉船等洲滩治理立项实施，提高重点地区、园区防洪标准。继续推进病险水库除险加固、中小河流治理和山洪灾害防治。强化管网支撑。支持“三横一纵”国家天然气输气管网和“环鄱阳湖”省级管网建设。坚持先规划后建设，先地下后地上，加强城市地下管网建设管理，加快污水管网和城市防洪排涝设施建设。

　　七是实施“协同共进行动”，着力形成统筹发展新格局。坚持区域协同、城乡统筹，在协调发展中拓展新空间。发展现代农业。优化农业产业布局，大力发展城郊蔬菜、鄱湖水产、西海林果、山区油茶和茶叶等产业，推进农业集群化发展。加快现代农业示范园区和环庐山、永武旅游休闲观光农业带建设，促进一二三产融合发展。构建新型农业经营体系，培育新型职业农民和农业经营主体，壮大龙头企业，提升农业规模化经营和社会化服务水平。加快农业标准化、信息化、品牌化建设，健全农产品质量安全体系，努力打造全国知名的绿色有机农产品供应基地。推动县域发展升级。坚持分类指导，实施权力下放，强化有效激励，促进县域经济整体提升、发展升级。力争“十三五”末，县县财政收入达20亿元，2个县（市、区）进入全省10强。促进城乡一体化。坚持以人的城镇化为核心，深化农村综合改革，促进农业转移人口进城落户。严格落实强农惠农政策，加快城镇村联动发展，加强农村环境整治，改善农村基础设施，建设和谐秀美乡村，努力形成以工促农、以城带乡、工农互惠、城乡一体的新型工农城乡关系。

　　八是实施“共建共享行动”，着力增强人民群众的幸福感和获得感。加快全面建成小康社会步伐，让人民群众共享经济发展成果，打造共建共享九江。坚持把脱贫攻坚作为“第一民生工程”，扎实推进十六项脱贫攻坚举措，打赢五场脱贫硬仗，确保到2018年实现建档立卡贫困人口全部脱贫、贫困村全部退出、贫困县提前“摘帽”。办好人民满意教育，基本实现教育现代化。繁荣发展文化事业，构建公共文化服务体系。推进健康九江建设，完善疾病预防控制和食品安全治理体系。坚持计划生育基本国策，促进人口均衡发展。加强公共体育设施建设，积极开展全民健身运动。实现更高质量的就业，形成合理的收入分配格局。健全基本养老保险和基本医疗保险制度，建立更加公平、更可持续的社会保障和救助体系。推进法治九江建设，完善信访调处机制，依法解决信访突出问题，提高社会治理法治化水平。推进平安九江建设，健全公共安全体系，坚决遏制重特大安全事故发生。完善立体化治安防控体系，确保人民安居乐业、社会和谐稳定。

　　三、2016年工作任务

　　2016年是“十三五”规划的开局之年，也是全面建成小康决胜阶段的首战之年。我们将认真贯彻中央经济工作会议精神，按照省委、省政府和市委的统一部署，坚持稳中求进的总基调，保持战略定力不动摇，着力稳定经济增长，着力深化改革开放，着力加快创新驱动，着力促进转型升级，着力保护生态环境，着力保障改善民生，加快“五大九江”建设，全面完成全年目标任务，确保实现“十三五”良好开局。

　　全市经济社会发展主要预期目标是：生产总值增长10%左右；财政总收入增长11%，地方公共预算收入增长11%；固定资产投资增长17%；社会消费品零售总额增长12%；居民消费价格总水平涨幅控制在2.5%；城镇居民和农村居民人均可支配收入分别增长10%和11%；完成省政府下达的节能减排任务。

　　为实现上述目标，我们将重点做好十个方面的工作：

　　（一）坚定不移主攻工业。打好“新工业十年行动”第一仗，全年实施亿元以上工业项目271个，总投资1448亿元，完成投资690亿元，力争规模以上工业增加值、主营业务收入和利税总额均增长10%。改造提升传统产业。加快企业技术改造，支持企业引进先进技术和装备，提高自动化、智能化生产水平，实施工业技改项目200个以上。加快延伸产业链条，重点支持九江石化等企业提高产品质量效益，鼓励引导赛得利化纤、德鑫纺织等企业优化产品结构。加快行业兼并重组，促进市场出清。培育壮大新兴产业。新能源产业，重点推进光伏发电应用、燃料乙醇和生物柴油项目；新材料产业，重点发展有机硅新材料、玻纤复合材料、多晶硅和金属新材料；电子信息产业，重点推进电子材料、电子元器件研发转型，加快形成平板显示、智能终端和集成电路等产业集群；节能电器产业，重点做大节能灯、空调和小家电等产业规模；生物医药产业，重点加强医药中间体技术研发，开发新型医药产品；绿色食品产业，重点做大粮油和水产品深加工产业，做优绿色饮品和休闲保健食品产业；装备制造产业，重点发展汽车及零部件，加快发展船用配套和海洋工程装备。支持卡博特气象白碳黑、中科鑫星新材料、盛祥电子等企业研发创新和成果转化。力争全年实施战略性新兴产业项目100个以上。促进产业集聚发展。支持龙头企业重组改造上下游配套企业，构建完善的产业链分工协作体系，形成“龙头企业顶天立地、配套企业铺天盖地”的格局。高标准建设石化产业园，延长石油加工产业链；依托星火有机硅产能扩张，做大有机硅“产业树”；依托九江发电厂、神华煤电，做大火电规模。帮扶企业做大做强。完善市领导挂点千亿产业、百亿企业和百部门帮百企等帮扶机制，实行重大工业项目全程代办服务。加强政银企对接，用好产业发展基金，帮助小企业做大、大企业做强。优化园区平台建设。完善园区企业信息、金融、中介、物流配送等配套服务，健全技术研发、质量检测等各类创新平台，支持九江经济技术开发区国家级产城融合示范区试点建设。提高园区投资强度，积极创建生态工业园区和循环经济园区。全面推进“两化融合”示范园区建设，力争示范企业达到100户以上。

　　（二）加快发展现代农业。促进农业升级。推进永久基本农田划定，落实粮食安全责任制，稳定粮食生产。加快“百县百园”建设，重点打造3-5个现代农业示范园。以茶叶、水产、油茶、珍珠等特色产业为重点，做大规模，打响品牌。发放“财政惠农信贷通”贷款10亿元，培育新型农业经营主体1000家，新增规模以上农业企业20家，创建“三品一标”和省级以上品牌50个。提升农村面貌。继续推进镇村联动建设，加强农民建房管理和风貌管控，抓好农村生活垃圾治理，完成87个中心村、830个新农村建设点村庄整治，新建改造农村公路1200公里，改造危桥50座。改善农民生活。大力推进“一村一品、一乡一业”富民工程，扶持一批种养、加工、餐饮、休闲养老、特色旅游等专业村庄建设，拓展农村二三产业增收空间。完成“新型职业农民培育”3000人，“一村一名大学生工程”招生800人，新增转移农村劳动力5.5万人。

　　（三）大力发展现代服务业。做旺文化旅游。加快庐山设市步伐，加强旅游资源整合和统一管理。建成庐山索道等项目，加快推进华夏国际旅游度假区、义门陈文化产业园等项目。组建文化旅游投资有限公司，启动浔阳古城保护和开发工作，实施庾亮南路历史文化街区改造。加快旅游标准化和智慧旅游建设，创新营销模式，提升从业人员素质，加大市场监管力度，支持庐山西海创建5A景区。力争全年旅游接待总人数、旅游总收入增长15%以上。做大现代金融。加大金融机构引进力度，完成农村信用社改制工作，新增村镇银行4家。拓展融资新品种，创新抵押担保方式，加大重点领域、重大项目信贷支持力度。加快完善证券、基金、保险、信托、投资等金融要素市场发展，引导非融资平台类企业在资本市场进行直接融资。鼓励企业参加信用评级，培育3-5家优质企业在银行间市场募集资金，扶持5家企业挂牌“新三板”。力争年末全市新增贷款260亿元。做活商贸物流。加快第三方物流和中小微物流企业发展，推进万达广场、京九商贸城、联盛快乐城等项目建设，打造一批国家级、省级特色商业街。加大农超对接力度，实施“快递下乡”工程，支持末端配送网络建设，引导农村邮政物流发展。支持共青城、恒盛科技园、横塘镇等电子商务示范基地建设，新增销售收入5000万元以上电商企业10家。

　　（四）提高新型城镇化水平。全年实施城建项目207个，总投资1037亿元，完成投资316亿元。高标准规划。推进“多规合一”，编制完成都市区总体规划和旧城功能区、濂溪片区、芳兰组团控制性详细规划，加快修编城市总体规划、历史文化名城保护规划，启动编制城市地下综合管廊、海绵城市、中心城区排水防涝和电力设施建设等规划。加强规划监管，做好城市设计。高品位建设。以八里湖中央商务区建设为重点，推动资源集中、要素集聚、业态集合，构建城市高端商圈，打造城市新地标。建设完成兴城南大道、中心城区高速进出口收费站“拆四建二”等项目，加快推进琴湖大道（南段）、第四自来水厂等续建项目，启动九江火车站外广场改造等项目，做好动物园迁建、八里湖综合管廊等前期工作。继续实施老城区边街小巷整治，推进小游园、公共绿地等建设。加快锁江楼、老京九市场等棚户区改造步伐，加大货币化安置力度，促进房地产去库存。高水平管理。巩固城市环境综合整治“攻坚年”成果，扎实开展“提升年”活动。加快推进市县两级城市管理改革，实施数字化城管二期工程。推行城市管理服务外包模式，提高环境卫生、园林绿化和设施管护等市场化管理水平。

　　（五）推进对内对外开放。突出招大引强。围绕重点产业，紧盯重点区域，创新招商方式，强化招商队伍，办好招商活动，提高招商实效。加强与国内、国外500强企业的对接，引进一批关联度大、带动性强、科技含量高的重大产业项目。力争市外5000万元以上项目实际进资增长20%以上，实际利用外资增长11%以上。加强区域合作。加快推进“昌九新区”建设，探索建立昌九扩大开放试验区，抱团融入长江中游城市群发展。推进沿江地区与宿松、望江、黄梅、武穴等地联动发展，推动建立“赣鄂皖沿江协作区”。积极对接上海自贸区，落实沪浔两地产业战略合作框架协议，支持彭泽“飞地”产业园建设。做优外经外贸。建立外贸综合服务平台，优化出口产品结构，实现进出口总额稳中有升，力争全年境外直接投资增长15%。完善口岸建设。完成九江电子口岸国际贸易“单一窗口”建设，协调推进九江港与南昌港一体化发展，加强九江港与上海外高桥码头、洋山港通关协作，提高九江港服务全省、辐射周边的能力。

　　（六）完善基础设施建设。建成一批。建成武九客专、修平高速、城东港区铁路专用线等交通项目，建成赤湖工业园、矶山化工园公用码头和神华、华中木业专业码头等港区项目，建成中电投江洲风电、赛城湖光伏等能源项目。完成105座小型水库除险加固和德安樟树水库新建项目。推进一批。加快推进九景衢铁路、合安九客专、昌九高速全线“四改八”、都九高速（都昌至星子段）等交通项目，加快推进红光作业区综合枢纽、疏港通道等港区项目，加快推进江西LNG天然气储配中心和3个220千伏、4个110千伏输变电项目，协调推进特高压入赣项目建设。谋划一批。启动昌九高铁、常岳九等铁路项目前期工作，启动宿九、瑞武等高速公路项目前期工作，启动湖口鄱阳湖、武穴至瑞昌码头等过江通道项目前期工作。

　　（七）加强生态环境保护。强化环境治理。全面落实大气、水体、土壤污染防治行动计划，深入实施治霾、减煤、除尘等专项行动。加强河道采砂管理，综合治理水土流失37万亩。开工建设白水湖和蛟滩污水处理厂，启动八赛连通控制闸工程。开展环保联动执法，严厉打击环境违法行为。注重生态建养。积极创建国家森林城市，推进浔南城市森林公园、庐山大道北段两侧退让绿化等项目建设，完成造林21.4万亩。加快实施柘林湖湖泊生态环保等项目，加强长江、修河、庐山西海等水生态保护。加快野生动植物救护中心和繁殖基地建设，支持星子湖湾建设国家湿地公园、武宁创建国家生态文明先行示范区。创新体制机制。健全河湖管理与保护制度，全面实行党政“一把手”负责的“河长制”。建立健全领导干部任期生态文明建设责任制度、自然资源资产离任审计制度和生态环境损害责任终身追究制。探索建立森林、湿地、矿产资源开发等生态补偿机制。倡导低碳生活，提高全民绿色环保意识。

　　（八）激发发展动力活力。加快重点领域改革。深化行政审批制度改革，推动“三单一网”建设，扩大政府购买服务试点目录和范围。完成行政机关公车改革，推动事业单位公车改革。深化财税体制改革，贯彻实施新预算法，实行预算公开、审计公开和财政预算绩效管理制度。推进医药卫生体制改革，启动城市公立医院改革。深化国资国企改革，加强经营性国有资产集中统一监管。加快不动产统一登记制度改革，完成农村土地承包经营权确权登记颁证工作，推进农村集体产权制度改革试点。增强科技创新能力。加大科技创新支持力度，完善科技投入保障机制，支持国内外科研院所、骨干企业在浔设立研发机构。实施“双百·双千”人才工程，推进院士工作站和人才安居工程建设，大力引进高端人才，积极培育本土人才。加强科技企业孵化器建设，新增省级以上高新技术企业20家。支持九江经济技术开发区、共青城市、庐山区等地高新技术产业园区建设。推动全民创业。落实小微企业支持政策，新增创业担保贷款11亿元。鼓励支持非公经济加快发展，引导社会资本参与基础设施和公用事业建设运营。支持共青城申报全国“双创”示范基地，支持都昌、修水等地返乡创业园建设。

　　（九）切实增进民生福祉。加大民生资金投入力度，提高财政性民生支出比重，精心办好一批民生实事，努力解决好群众普遍关心的热点难点问题。抓好扶贫开发。扶持贫困村、贫困户发展致富产业、就地创业，减少贫困人口9.1万人，完成扶贫移民搬迁1万人，非重点县贫困村、贫困人口全部退出。实施精准特惠救助扶贫，统筹贫困地区一体化发展，完成333个贫困自然村整治。做实民生工程。加大棚户区改造力度，创新保障房建设管理运营模式，继续实施农村困难群众危房改造工程。促进创业就业，新增城镇就业5.4万人，为城乡困难群众购买公益性就业岗位4400个。建立健全“放心粮油”与应急供应网络，新建、改造“农超对接”网点10家。加快中心城区公交场站改造，新建公共停车场6个，续建、改造农贸市场12家，建成社区居家养老服务中心13家。继续推进重大疾病免费救治、新农合大病保险、医疗责任险、疾病应急救助等工作。完善医疗救助、临时救助、保险救治等救助体系，不断提高低保、五保救助水平。办理法律援助案件2000件。发展社会事业。加快乡镇公办幼儿园建设，扶持普惠性民办幼儿园发展；完成农村义务教育学校标准化建设，做好农民工子女随迁入学工作；加快普及高中阶段教育，推动中等职业学校达标，加快共青科教城和高等职业教育实训基地建设，促进高等教育内涵式发展，努力办好特殊教育。推进国家公共文化服务体系示范区创建工作，开展农村文化三项活动。加强公共卫生服务能力建设，做好重大传染病防治工作，提升职业健康工作水平，促进基本公共卫生计生服务均等化。依法实施“全面两孩”生育政策，做好关心下一代工作。扎实开展第三次全国农业普查。办好市第十二届运动会。加强国防后备力量建设，深入开展“双拥”创建活动，支持部队改革，促进军民融合深度发展。充分发挥统计、审计、物价、工商、质监、供销、国安、消防、防震减灾、应急管理、人民防空、盐业、邮政、海事、气象、水文、民族宗教、外事侨务、涉台事务、驻外联络、新闻出版、广播影视、档案、地方志、保密、机构编制、无线电管理、妇女儿童、老龄、残疾人、红十字、慈善等工作在经济发展和社会建设中的促进作用。维护和谐稳定。严厉打击各种违法犯罪活动，不断提升公众安全感。启动“七五”普法。加大社会治安综合治理力度，加强社会矛盾排查，畅通信访渠道，依法有效预防和化解社会矛盾。严格落实安全生产责任制，深入开展隐患排查治理，做好生产安全、城市安全、食品安全、交通安全、森林防火等工作，保障人民群众生命财产安全。

　　（十）加强政府自身建设。按照“法治、为民、诚信、高效、廉洁”的要求，打造实在实干政府，切实增强政府执行力和公信力。打造法治政府。依法健全重大行政决策制度，促进决策的科学化、民主化和规范化。全面落实行政执法责任制和责任追究制度，规范行政执法行为。做好城乡建设与管理、环境和历史文化保护等领域立法工作。自觉接受人大及其常委会的法律监督、工作监督和政协的民主监督，认真办理人大代表议案、建议和政协委员提案，广泛听取民主党派、工商联、老同志和社会各界人士的意见、建议。打造为民政府。坚持以人为本、执政为民，努力办群众满意的事情。坚决纠正、查处行政不作为、乱作为和损害群众利益的不正之风及腐败问题，切实维护群众利益。积极开展企业、客商评议部门活动，完善群众诉求表达长效机制，解决群众的合理诉求。打造诚信政府。保持政策的连续性和稳定性，做到言而有信、信守承诺。推动诚信体系建设，完善信用信息网络平台，加强政府工作人员诚信管理和教育，以诚信办事、诚信服务取信于民，通过政务诚信示范引领全社会诚信建设。打造高效政府。加强政府系统公务员队伍建设，倡导立说立行、即说即办的作风，强化敢于负责、勇于担当的意识，切实提高政府办事效能。全面落实首问负责制、限时办结制和责任追究制，确保政令畅通。坚持守土有责、合力推进，强化政府各级、各部门间的协调配合。规范行政审批程序，强化跟踪督办和效能问责，确保各项工作落到实处。打造廉洁政府。坚持把纪律和规矩挺在前面，全面落实党风廉政建设责任制，模范遵守廉洁自律各项要求，严格落实“一岗双责”“一案双查”。巩固“三严三实”专题教育成果，坚决落实“八项规定”，驰而不息反对“四风”，严控“三公”经费和一般性开支。健全行政监察、审计监督、财政监督机制，强化工程建设、政府采购、资金使用、公共资源和产权交易等重点领域和重要环节的监管，着力营造风清气正的政治生态。

　　各位代表！新常态赋予新使命，新征程承载新梦想。让我们在省委、省政府和市委的正确领导下，凝心聚力，团结拼搏，真抓实干，为加快建设“五大九江”、推动“双核”发展、全面建成小康而努力奋斗！
